# Risk Factors Associated With Non-Respondence to Methotrexate in Rheumatoid Arthritis Patients

**DOI:** 10.7759/cureus.18112

**Published:** 2021-09-20

**Authors:** Aman Siddiqui, Ali Totonchian, Jamila Begum Jabar Ali, Ishtiaq Ahmad, Jai Kumar, Sheena Shiwlani, Daniya Muhammad Haroon, Neeraj Makheja, Amber Rizwan

**Affiliations:** 1 Internal Medicine, Dow University of Health Sciences, Civil Hospital Karachi, Karachi, PAK; 2 Internal Medicine, Dow University of Health Sciences, Dow International Medical College, Karachi, PAK; 3 Internal Medicine, California Institute of Behavioral Neurosciences and Psychology, Fairfield, USA; 4 Internal Medicine, Khyber Medical College, Peshawar, PAK; 5 Internal Medicine, Liaquat University of Medical and Health Sciences, Jamshoro, PAK; 6 Internal Medicine, Isra University Hospital, Hyderabad, PAK; 7 Internal Medicine, Bahria University Medical and Dental College, Karachi, PAK; 8 Internal Medicine, Ghulam Muhammad Mahar Medical College, Karachi, PAK; 9 Family Medicine, Jinnah Post Graduate Medical Center, Karachi, PAK

**Keywords:** autoimmune, rheumatoid factor, ra, methotrexate, rheumatoid arthritis

## Abstract

Introduction: Oral methotrexate (MTX) is the first-line therapy for patients with rheumatoid arthritis (RA). However, not all RA patients respond to MTX. In this study, we will determine the risk factors associated with MTX failure.

Methods: This retrospective study was conducted in tertiary care hospital in Pakistan. Data of 612 patients who were diagnosed with RA from June 2019 to January 2021 were retrieved from the medical record room. After inclusion, patients were divided into two groups; respondent and non-respondent. Their characteristics and demographics were compared.

Results: Out of the total 612 patients, 112 (18.3%) were labelled as non-respondent to MTX. Non-respondents had a higher predominance of females (86.6% vs. 60.2%; p-value: 0.001), participants with body mass index (BMI) >25 kg/m^2^ (54.4% vs. 22.4%; p-value: <0.00001), smokers (34.8% vs. 18.2%; p-value: 0.0001), participants with diabetes (47.3% vs. 23.4%; p-value: <0.0001) and rheumatoid factor positivity (91.0% vs. 64.8%; p-value: <0.0001).

Conclusion: Female gender, higher BMI, smoking, higher disease activity, and diabetes were associated with MTX failure. These easily available parameters can help predict the disease process and outcome of treatment. It is important to screen patients who are at risk of MTX failure, so a contingent treatment plan can be devised, in case patients do not respond to MTX.

## Introduction

Rheumatoid arthritis (RA) is an inflammatory autoimmune disease with multisystem involvement. It is characterized mainly by musculoskeletal pain, swollen and stiff joints that can severely handicap and have a negative impact on the quality of life [1]. Hence, its diagnosis and proper management are crucial. The global estimated prevalence of RA is 0.5-1% of the population and ranks next to osteoarthritis and gout as major causes of disability [2].

Nonsteroidal anti-inflammatory drugs (NSAIDs) are used for the symptomatic management of pain and inflammation; whereas the first-line therapy involves the use of disease-modifying anti-rheumatic drugs (DMARDs) for all the newly diagnosed cases of RA [3]. Other therapies include biological-response modifiers and agents that selectively inhibit specific molecules of the immune system [3]. Oral methotrexate (MTX) is deemed as the first-line conventional therapy for patients with RA as it is cheap and efficacious. It can be prescribed alone or in combination with other agents, including biological agents. However, its discontinuation within 12 months is observed in approximately one-quarter of patients [4] and little is known regarding the possibility of prediction of patients’ response to treatment.

Owing to the very limited data available on risk factors associated with MTX failure in RA patients, particularly in the South Asian region, we aim to determine the risk factors associated with MTX failure in patients with RA in this study.

## Materials and methods

This retrospective study was conducted in a tertiary care hospital in Pakistan. Data of 612 patients who were diagnosed with RA from June 2019 to January 2021 were retrieved from the medical record room after approval from the Institutional Review Board of Dow University of Health Sciences (DUHS/IRB/2019/16-Revised). All patients with a diagnosis of RA with complete records were enrolled in the study. The complete record was defined as the availability of parameters of the participants, including age, gender, body mass index (BMI), smoking status, rheumatoid factor (RF) status, the value of anti-citrullinated protein, presence of hypertension and diabetes, and if the patient responded to MTX. Dose for methotrexate was 10 mg daily. Patients in whom dose adjustment of MTX was done, were not included in study. Patients with chronic liver disease and chronic kidney disease were also excluded from the study.

After inclusion, patients were divided into two groups; respondent and non-respondent. Patients response was first measured six months after starting MTX. Non-respondents were defined using the European League against Rheumatism (EULAR) response criteria [5]. Characteristics and demographics of both groups were compared. The obtained data were analyzed using the Statistical Packages for the Social Sciences, version 22.0 (SPSS, IBM Corporation, Armonk, New York, USA). Descriptive analysis of continuous variables was done and presented as mean and standard deviation (SD). The categorical variables were expressed by percentages and frequencies. Chi-square and t-test were used as appropriate. A p-value of less than 0.05 was considered as the level of significance.

## Results

A majority of patients responded to MTX, whereas 112 (18.3%) participants were labelled as non-respondent to MTX. Non-respondents had a higher predominance of females (86.6% vs. 60.2%; p-value: 0.001), participants with BMI >25 kg/m^2^ (54.4% vs. 22.4%; p-value: <0.00001), smokers (34.8% vs. 18.2%; p-value: 0.0001), participants with diabetes (47.3% vs. 23.4%; p-value: <0.0001) RF positivity (91.0% vs. 64.8%; p-value: <0.0001; Table 1).

**Table 1:** Comparison of characteristics and risk factors of participants of both groups BMI: body mass index, CCP: cyclic citrullinated peptide, kg/m^2^: kilograms per square meter, NS: nonsignificant, RF: rheumatoid factor, µ/mL: units per milliliters.

Risk factors	Respondents (n=500)	Non-respondents (n=112)	p-value
Age at diagnosis (in years)	42 ± 6	41 ± 7	NS
Female (%)	362 (60.2%)	97 (86.6%)	0.001
BMI more than 25 kg/m^2^	112 (22.4%)	61 (54.4%)	<0.00001
Smoking (%)	91 (18.2%)	39 (34.8%)	0.0001
Hypertension (%)	101 (20.2%)	25 (22.3%)	NS
Diabetes (%)	117 (23.4%)	53 (47.3%)	<0.0001
Positive RF	324 (64.8%)	102 (91.0%)	<0.0001
Anti-CCP at admission (µ/mL)	30 ± 7	33 ± 8	NS

## Discussion

Our study found that female gender, higher BMI, smoking, positive RF status, and diabetes are the risk factors that may be responsible for RA patients not responding to MTX. The results of our study are consistent with that of Bluett et al., stating the association of MTX failure in patients with RF positivity, the onset of symptoms at a younger age, and higher baseline disease activity (DAS-28) [4]. A Swedish pharmacotherapy trial (SWEFOT) also suggested that female sex, high BMI, smoking, and functional impairment were linked strongly with a low probability of achieving remission or better clinical outcomes [6]. A number of studies support the association of early MTX failure in the female gender secondary to inefficacy [7]. Owing to the higher baseline disease activity in women and the impact of hormones on the metabolism of MTX, women tend to be less likely to respond to MTX [4,8].

Another risk factor associated with MTX failure in our study was obesity. According to the SWEFOT trial, obesity was the strongest independent predictor of non-remission after two years of treatment [6]. Another study including 83% of patients on MTX concluded a statistically significant dose-response relationship between BMI and good clinical outcomes and remission [9]. In comparison to the normal BMI counterparts, patients with BMI ≥25 kg/m^2^ were found to have 51% and 42% lower odds of achieving low disease activity and remission, respectively [9]. Besides increased secretion of pro-inflammatory signals such as TNF-α and interleukin-6 from adipose tissue [10], adiponectin and leptin are other biomarkers produced from adipose tissue. Adiponectin leads to bone erosions while leptin induces inflammation [6]. Hence, obesity is associated with higher levels of inflammatory markers than normal-weight individuals [11].

Smoking is not only a risk factor for RA, but also enhances the basal metabolic rate which in turn may affect MTX metabolism, causing a reduced response in RA patients [12]. Therefore, smoking cessation is important in RA patients in order to have a maximum clinical response to MTX. Ruscitti et al. found a close association between uncontrolled disease activity and deranged glucose metabolism, establishing a link between diabetes and poor response to MTX [13]. Furthermore, consistent with our study, several other studies have reported RF positivity as an independent risk factor for earlier MTX failure [4,7]. However, Varatharajan et al. demonstrated that patients with seronegative arthritis were more likely to cease MTX therapy for all causes [14]. Researches support the association of younger age with increased MTX failure [6,8]; however, we found no significant association between age and poor response to MTX.

Despite MTX is suggested as the first DMARD of choice for RA worldwide [15], its efficacy is not universal and discontinuation is seen by a significant proportion of patients secondary to either inefficacy or adverse outcomes. Hence, it is of prime importance to predict treatment outcomes in patients unlikely to respond to MTX. Patients who fail treatment with MTX can be switched to other DMARDs from which they would be more likely to derive benefit [8].

To the best of our knowledge, this is the first study in a regional setting that discusses the risk factors associated with MTX failure in RA patients. There were certain limitations as well. First, since the study was conducted in a single institute, the sample size was limited and lacked diversity. Hence, care should be taking while inferring the result to the larger population. Second, since it was a retrospective study, not all variables and risk factors were available for analysis. Further, large-scale studies are needed to assess the risk factors associated with MTX failure in the regional South Asian population.

## Conclusions

Female gender, higher BMI, smoking, higher disease activity, and diabetes were associated with MTX failure. These easily available parameters can help predict the disease process and outcome of treatment. This study highlights the importance of lifestyle modification as a cornerstone of management and remission of RA. It is important to screen patients who are at risk of MTX failure, so a contingent treatment plan can be devised, in case patients do not respond to MTX. This will allow appropriate management of RA and reduce the risk of complications.
